# Redescription of *Mesoschendylajavanica* (Attems, 1907) and its first records from Borneo (Chilopoda, Geophilomorpha, Schendylidae)

**DOI:** 10.3897/BDJ.13.e156917

**Published:** 2025-05-23

**Authors:** George Popovici, Nesrine Akkari, Gregory D. Edgecombe

**Affiliations:** 1 Imperial College London, London, United Kingdom Imperial College London London United Kingdom; 2 ”Grigore Antipa” National Museum of Natural History, Bucharest, Romania ”Grigore Antipa” National Museum of Natural History Bucharest Romania; 3 Natural History Museum Vienna, Vienna, Austria Natural History Museum Vienna Vienna Austria; 4 Natural History Museum, London, United Kingdom Natural History Museum London United Kingdom

**Keywords:** centipede, Sarawak, East Malaysia, biogeography

## Abstract

**Background:**

The geophilomorph *Mesoschendylajavanica* (Attems, 1907) was originally described from a small number of males collected from bat guano in Tjompea (Ciampea), Java. Subsequently, no additional material was identified. The type series remained the only specimens belonging to this genus known from Asia.

**New information:**

*Mesoschendylajavanica* is re-discovered 118 years after its original description amongst centipedes collected from soil cores taken during the 1977–1978 Royal Geographical Society Gunung Mulu Expedition to Sarawak (Borneo, East Malaysia) and is deposited in the collection of the Natural History Museum (London). The new material comprising 49 specimens, amongst which are the first known females, is described and illustrated, shedding light on intraspecific morphological variation. The syntypes and sole previously available specimens are redescribed and illustrated, completing the summary original description of the species. Ecological and biogeographical notes are provided for Sarawak specimens.

## Introduction

The centipede fauna of the Indo-Malayan Region remains incompletely known despite a long history of taxonomic research (Haase 1887, Attems 1907, Attems 1914, Verhoeff 1937). Although a principal subset of soil arthropod macrofauna across a range of habitat types in this biogeographic region (Collins 1980), centipedes remain poorly known from ecological and biogeographical standpoints due to the patchy nature of historical collections and the paucity of habitat and locality data included in past taxonomic treatments. This knowledge gap is particularly salient for small (< 20 mm) soil centipedes (Geophilomorpha), which are frequently overlooked or inaccessible when hand collecting in soil-surface microhabitats (Baba et al. 2023). Many geophilomorph species named from the Sunda Islands in the first half of the 20^th^ century remain poorly characterised as they were described from a small number of specimens, frequently only one and have not been subsequently re-discovered (Table 1).

*Mesoschendylajavanica* (Attems, 1907) was described from a few specimens collected from bat guano at Tjompea (Ciampea), West Java and originally assigned to the genus *Schendyla* Bergsøe and Meinert, 1866. Subsequent revision (Attems 1929) placed the species under *Mesoschendyla* Attems, 1909, on the basis of the mandibular dentate lamella not having distinct blocks of teeth, sternal pore fields being confined to the anterior part of the trunk and a single, large pair of coxal pores on the last leg-bearing segment. All of the other eight known species of *Mesoschendyla* have been recorded from mainland Africa and Madagascar (reviewed and keyed by Crabill (1968)) and no new *Mesoschendyla* records from Asia have been published since the original description of *M.javanica*.

We identify 49 specimens of *M.javanica* in soil core samples taken from Gunung Mulu National Park, Sarawak, Borneo (East Malaysia) during the 1977–1978 Royal Geographical Society expedition to this region. Their identity is confirmed by comparison to the syntypes of *M.javanica*, allowing for a detailed redescription of the species, based on specimens collected in Sarawak and micropreparations of the syntypes, including the first description of the female. Habitat data are provided for all new localities in Borneo with subsequent discussion of the ecology and biogeography of *M.javanica* in the Indo-Malayan Region.

## Materials and methods

Specimens from Sarawak, Malaysia, were examined with a Nikon SMZ 1270 stereomicroscope and a Leica DMR binocular microscope and camera lucida drawings were created with the aid of a drawing tube. All examined specimens from the Royal Geographical Society 1977–1978 expedition to Gunung Mulu, Sarawak, are deposited in the NHMUK (London) collection, with collection data provided below. Locality data provided refer to the sample sites documented by previously published proceedings of the expedition (Collins 1980, Anderson et al. 1983, Proctor et al. 1983).

The remaining type material of *M.javanica*, deposited in the NHMW collection, comprises two slide-mounted specimens which were photographed with a Nikon Ds-Ri2 camera mounted on a Nikon Eclipse Ni compound microscope, to complete the morphological description of the species. An overview of the slides was photographed using a Nikon D7200 with a Nikkor 60 mm 1:2.8 G ED macro lens mounted on a Stand System Kaiser RTX 2 XA with two cold light lamps (Kaiser RB 218N HF). Morphological descriptions of the syntypes are included below in square brackets (“[]”).

The distribution map of *M.javanica* records was created with QGIS version 3.40.5.

## Taxon treatments

### Mesoschendyla javanica

(Attems, 1907)

159ED58B-C85D-5153-877C-D55F6F474323

#### Materials

- **Type status:** Other material. **Occurrence:** catalogNumber: NHMUK015991403; recordedBy: J. G. E. Lewis; individualCount: 1; sex: male; lifeStage: adult; preparations: preserved in 70% ethanol; occurrenceID: 6B8685CF-EE92-586C-98F7-C0DB61E3EB40; **Taxon:** scientificName: Mesoschendylajavanica; kingdom: Animalia; phylum: Arthropoda; class: Chilopoda; order: Geophilomorpha; family: Schendylidae; genus: Mesoschendyla; specificEpithet: javanica; taxonRank: species; scientificNameAuthorship: (Attems, 1907); taxonomicStatus: accepted; **Location:** continent: Asia; islandGroup: Greater Sunda Islands; island: Borneo; country: Malaysia; countryCode: MY; stateProvince: Sarawak; locality: Gunung Mulu National Park; verbatimLocality: Gunung Mulu National Park; verbatimElevation: 500 m; locationRemarks: humus in mixed dipterocarp forest; verbatimCoordinates: 4°02'38"N 114°52'13"E; decimalLatitude: 4.043889; decimalLongitude: 114.870278; georeferenceProtocol: label; **Identification:** identifiedBy: George Popovici; dateIdentified: 03-2025; **Event:** samplingProtocol: hand collection; eventDate: 01-08-1978; year: 1978; month: 8; day: 1; fieldNumber: C49; **Record Level:** collectionID: urn:lsid:biocol.org:col:15660; institutionCode: NHMUK; collectionCode: Arachnida and Myriapoda; ownerInstitutionCode: NHMUK; basisOfRecord: PreservedSpecimen
- **Type status:** Other material. **Occurrence:** catalogNumber: NHMUK015991357; recordedBy: H. W. Vallack; individualCount: 3; sex: female; lifeStage: adult; preparations: preserved in 70% ethanol; occurrenceID: 254DBD01-71A9-5980-B727-E8DE4531AD7C; **Taxon:** scientificName: Mesoschendylajavanica; kingdom: Animalia; phylum: Arthropoda; class: Chilopoda; order: Geophilomorpha; family: Schendylidae; genus: Mesoschendyla; specificEpithet: javanica; taxonRank: species; scientificNameAuthorship: (Attems, 1907); taxonomicStatus: accepted; **Location:** continent: Asia; islandGroup: Greater Sunda Islands; island: Borneo; country: Malaysia; countryCode: MY; stateProvince: Sarawak; locality: Gunung Mulu National Park; verbatimLocality: Gunung Mulu National Park; locationRemarks: soil cores; georeferenceProtocol: label; **Identification:** identifiedBy: George Popovici; dateIdentified: 03-2025; **Event:** samplingProtocol: soil core sample; eventDate: 1978; year: 1978; **Record Level:** collectionID: urn:lsid:biocol.org:col:15660; institutionCode: NHMUK; collectionCode: Arachnida and Myriapoda; ownerInstitutionCode: NHMUK; basisOfRecord: PreservedSpecimen
- **Type status:** Other material. **Occurrence:** catalogNumber: NHMUK015991357; recordedBy: H. W. Vallack; individualCount: 1; lifeStage: juvenile; preparations: preserved in 70% ethanol; occurrenceID: 9E4F1AEA-7C0E-5872-A622-9571FEB8B241; **Taxon:** scientificName: Mesoschendylajavanica; kingdom: Animalia; phylum: Arthropoda; class: Chilopoda; order: Geophilomorpha; family: Schendylidae; genus: Mesoschendyla; specificEpithet: javanica; taxonRank: species; scientificNameAuthorship: (Attems, 1907); taxonomicStatus: accepted; **Location:** continent: Asia; islandGroup: Greater Sunda Islands; island: Borneo; country: Malaysia; countryCode: MY; stateProvince: Sarawak; locality: Gunung Mulu National Park; verbatimLocality: Gunung Mulu National Park; locationRemarks: soil cores; georeferenceProtocol: label; **Identification:** identifiedBy: George Popovici; dateIdentified: 03-2025; **Event:** samplingProtocol: soil core sample; eventDate: 1978; year: 1978; **Record Level:** collectionID: urn:lsid:biocol.org:col:15660; institutionCode: NHMUK; collectionCode: Arachnida and Myriapoda; ownerInstitutionCode: NHMUK; basisOfRecord: PreservedSpecimen
- **Type status:** Other material. **Occurrence:** catalogNumber: NHMUK015991357; recordedBy: H. W. Vallack; individualCount: 1; sex: male; lifeStage: adult; preparations: preserved in 70% ethanol; occurrenceID: 378B75DD-3C2A-5104-93E6-721BA6208A08; **Taxon:** scientificName: Mesoschendylajavanica; kingdom: Animalia; phylum: Arthropoda; class: Chilopoda; order: Geophilomorpha; family: Schendylidae; genus: Mesoschendyla; specificEpithet: javanica; taxonRank: species; scientificNameAuthorship: (Attems, 1907); taxonomicStatus: accepted; **Location:** continent: Asia; islandGroup: Greater Sunda Islands; island: Borneo; country: Malaysia; countryCode: MY; stateProvince: Sarawak; locality: Gunung Mulu National Park; verbatimLocality: Gunung Mulu National Park; locationRemarks: soil cores; georeferenceProtocol: label; **Identification:** identifiedBy: George Popovici; dateIdentified: 03-2025; **Event:** samplingProtocol: soil core sample; eventDate: 1978; year: 1978; **Record Level:** collectionID: urn:lsid:biocol.org:col:15660; institutionCode: NHMUK; collectionCode: Arachnida and Myriapoda; ownerInstitutionCode: NHMUK; basisOfRecord: PreservedSpecimen
- **Type status:** Other material. **Occurrence:** catalogNumber: NHMUK015991355; recordedBy: N. M. Collins; individualCount: 1; sex: male; lifeStage: adult; preparations: preserved in 70% ethanol; occurrenceID: 65A79BF1-8BC5-5D76-AF5F-E8A1D16B22A4; **Taxon:** scientificName: Mesoschendylajavanica; kingdom: Animalia; phylum: Arthropoda; class: Chilopoda; order: Geophilomorpha; family: Schendylidae; genus: Mesoschendyla; specificEpithet: javanica; taxonRank: species; scientificNameAuthorship: (Attems, 1907); taxonomicStatus: accepted; **Location:** continent: Asia; islandGroup: Greater Sunda Islands; island: Borneo; country: Malaysia; countryCode: MY; stateProvince: Sarawak; locality: Gunung Mulu National Park; verbatimLocality: Gunung Mulu National Park; verbatimElevation: 1650 m; locationRemarks: altitude zonation site H, lower montane forest soil cores; verbatimCoordinates: 4°02'19"N 114°54'13"E; decimalLatitude: 4.038611; decimalLongitude: 114.903611; georeferenceProtocol: label; **Identification:** identifiedBy: George Popovici; dateIdentified: 03-2025; **Event:** samplingProtocol: soil core sample; eventDate: 19/20-02-1978; year: 1978; month: 2; day: 19–20; **Record Level:** collectionID: urn:lsid:biocol.org:col:15660; institutionCode: NHMUK; collectionCode: Arachnida and Myriapoda; ownerInstitutionCode: NHMUK; basisOfRecord: PreservedSpecimen
- **Type status:** Other material. **Occurrence:** catalogNumber: NHMUK015991354; recordedBy: N. M. Collins; individualCount: 1; sex: male; lifeStage: adult; preparations: preserved in 70% ethanol; occurrenceID: EAE88063-CA6E-5EBD-9497-62F639263596; **Taxon:** scientificName: Mesoschendylajavanica; kingdom: Animalia; phylum: Arthropoda; class: Chilopoda; order: Geophilomorpha; family: Schendylidae; genus: Mesoschendyla; specificEpithet: javanica; taxonRank: species; scientificNameAuthorship: (Attems, 1907); taxonomicStatus: accepted; **Location:** continent: Asia; islandGroup: Greater Sunda Islands; island: Borneo; country: Malaysia; countryCode: MY; stateProvince: Sarawak; locality: Gunung Mulu National Park; verbatimLocality: Gunung Mulu National Park; verbatimElevation: 200–250 m; locationRemarks: mixed dipterocarp forest soil cores; verbatimCoordinates: 4°03'00"N 114°51'40"E; decimalLatitude: 4.05; decimalLongitude: 114.861111; georeferenceProtocol: label; **Identification:** identifiedBy: George Popovici; dateIdentified: 03-2025; **Event:** samplingProtocol: soil core sample; eventDate: 12/14-12-1978; year: 1978; month: 12; day: 12–14; **Record Level:** collectionID: urn:lsid:biocol.org:col:15660; institutionCode: NHMUK; collectionCode: Arachnida and Myriapoda; ownerInstitutionCode: NHMUK; basisOfRecord: PreservedSpecimen
- **Type status:** Other material. **Occurrence:** catalogNumber: NHMUK015991354; recordedBy: N. M. Collins; individualCount: 1; sex: female; lifeStage: adult; preparations: preserved in 70% ethanol; occurrenceID: 30269743-D194-5AB7-B0E8-981737646CD2; **Taxon:** scientificName: Mesoschendylajavanica; kingdom: Animalia; phylum: Arthropoda; class: Chilopoda; order: Geophilomorpha; family: Schendylidae; genus: Mesoschendyla; specificEpithet: javanica; taxonRank: species; scientificNameAuthorship: (Attems, 1907); taxonomicStatus: accepted; **Location:** continent: Asia; islandGroup: Greater Sunda Islands; island: Borneo; country: Malaysia; countryCode: MY; stateProvince: Sarawak; locality: Gunung Mulu National Park; verbatimLocality: Gunung Mulu National Park; verbatimElevation: 200–250 m; locationRemarks: mixed dipterocarp forest soil cores; verbatimCoordinates: 4°03'00"N 114°51'40"E; decimalLatitude: 4.05; decimalLongitude: 114.861111; georeferenceProtocol: label; **Identification:** identifiedBy: George Popovici; dateIdentified: 03-2025; **Event:** samplingProtocol: soil core sample; eventDate: 12/14-12-1978; year: 1978; month: 12; day: 12–14; **Record Level:** collectionID: urn:lsid:biocol.org:col:15660; institutionCode: NHMUK; collectionCode: Arachnida and Myriapoda; ownerInstitutionCode: NHMUK; basisOfRecord: PreservedSpecimen
- **Type status:** Other material. **Occurrence:** catalogNumber: NHMUK015991353; recordedBy: N. M. Collins; individualCount: 4; sex: male; lifeStage: adult; preparations: preserved in 70% ethanol; occurrenceID: FEDCF743-60CE-5BEE-ACB6-EC7F62B45404; **Taxon:** scientificName: Mesoschendylajavanica; kingdom: Animalia; phylum: Arthropoda; class: Chilopoda; order: Geophilomorpha; family: Schendylidae; genus: Mesoschendyla; specificEpithet: javanica; taxonRank: species; scientificNameAuthorship: (Attems, 1907); taxonomicStatus: accepted; **Location:** continent: Asia; islandGroup: Greater Sunda Islands; island: Borneo; country: Malaysia; countryCode: MY; stateProvince: Sarawak; locality: Gunung Mulu National Park; verbatimLocality: Gunung Mulu National Park; verbatimElevation: 1930 m; locationRemarks: altitude zonation site J, upper montane forest soil cores; verbatimCoordinates: 4°02'46"N 114°55'06"E; decimalLatitude: 4.046111; decimalLongitude: 114.918333; georeferenceProtocol: label; **Identification:** identifiedBy: George Popovici; dateIdentified: 03-2025; **Event:** samplingProtocol: soil core sample; eventDate: 19/20-03-1978; year: 1978; month: 3; day: 19–20; **Record Level:** collectionID: urn:lsid:biocol.org:col:15660; institutionCode: NHMUK; collectionCode: Arachnida and Myriapoda; ownerInstitutionCode: NHMUK; basisOfRecord: PreservedSpecimen
- **Type status:** Other material. **Occurrence:** catalogNumber: NHMUK015991353; recordedBy: N. M. Collins; individualCount: 2; sex: female; lifeStage: adult; preparations: preserved in 70% ethanol; occurrenceID: 9088387F-E557-58F6-A404-F2F3CB88A496; **Taxon:** scientificName: Mesoschendylajavanica; kingdom: Animalia; phylum: Arthropoda; class: Chilopoda; order: Geophilomorpha; family: Schendylidae; genus: Mesoschendyla; specificEpithet: javanica; taxonRank: species; scientificNameAuthorship: (Attems, 1907); taxonomicStatus: accepted; **Location:** continent: Asia; islandGroup: Greater Sunda Islands; island: Borneo; country: Malaysia; countryCode: MY; stateProvince: Sarawak; locality: Gunung Mulu National Park; verbatimLocality: Gunung Mulu National Park; verbatimElevation: 1930 m; locationRemarks: altitude zonation site J, upper montane forest soil cores; verbatimCoordinates: 4°02'46"N 114°55'06"E; decimalLatitude: 4.046111; decimalLongitude: 114.918333; georeferenceProtocol: label; **Identification:** identifiedBy: George Popovici; dateIdentified: 03-2025; **Event:** samplingProtocol: soil core sample; eventDate: 19/20-03-1978; year: 1978; month: 3; day: 19–20; **Record Level:** collectionID: urn:lsid:biocol.org:col:15660; institutionCode: NHMUK; collectionCode: Arachnida and Myriapoda; ownerInstitutionCode: NHMUK; basisOfRecord: PreservedSpecimen
- **Type status:** Other material. **Occurrence:** catalogNumber: NHMUK015991353; recordedBy: N. M. Collins; individualCount: 1; lifeStage: juvenile; preparations: preserved in 70% ethanol; occurrenceID: 13BB87FE-F58E-5404-9A4B-8ECC4ACDA55C; **Taxon:** scientificName: Mesoschendylajavanica; kingdom: Animalia; phylum: Arthropoda; class: Chilopoda; order: Geophilomorpha; family: Schendylidae; genus: Mesoschendyla; specificEpithet: javanica; taxonRank: species; scientificNameAuthorship: (Attems, 1907); taxonomicStatus: accepted; **Location:** continent: Asia; islandGroup: Greater Sunda Islands; island: Borneo; country: Malaysia; countryCode: MY; stateProvince: Sarawak; locality: Gunung Mulu National Park; verbatimLocality: Gunung Mulu National Park; verbatimElevation: 1930 m; locationRemarks: altitude zonation site J, upper montane forest soil cores; verbatimCoordinates: 4°02'46"N 114°55'06"E; decimalLatitude: 4.046111; decimalLongitude: 114.918333; georeferenceProtocol: label; **Identification:** identifiedBy: George Popovici; dateIdentified: 03-2025; **Event:** samplingProtocol: soil core sample; eventDate: 19/20-03-1978; year: 1978; month: 3; day: 19–20; **Record Level:** collectionID: urn:lsid:biocol.org:col:15660; institutionCode: NHMUK; collectionCode: Arachnida and Myriapoda; ownerInstitutionCode: NHMUK; basisOfRecord: PreservedSpecimen
- **Type status:** Other material. **Occurrence:** catalogNumber: NHMUK015991352; recordedBy: N. M. Collins; individualCount: 1; sex: male; lifeStage: adult; preparations: preserved in 70% ethanol; occurrenceID: FA4B8ECB-78E2-5F1F-BFC2-C2FA04A2AB9E; **Taxon:** scientificName: Mesoschendylajavanica; kingdom: Animalia; phylum: Arthropoda; class: Chilopoda; order: Geophilomorpha; family: Schendylidae; genus: Mesoschendyla; specificEpithet: javanica; taxonRank: species; scientificNameAuthorship: (Attems, 1907); taxonomicStatus: accepted; **Location:** continent: Asia; islandGroup: Greater Sunda Islands; island: Borneo; country: Malaysia; countryCode: MY; stateProvince: Sarawak; locality: Gunung Mulu National Park; verbatimLocality: Gunung Mulu National Park; verbatimElevation: 2376 m; locationRemarks: altitude zonation site L, upper montane forest soil core; verbatimCoordinates: 4°02'43"N 114°55'47"E; decimalLatitude: 4.045278; decimalLongitude: 114.929722; georeferenceProtocol: label; **Identification:** identifiedBy: George Popovici; dateIdentified: 03-2025; **Event:** samplingProtocol: soil core sample; eventDate: 18-03-1978; year: 1978; month: 3; day: 18; **Record Level:** collectionID: urn:lsid:biocol.org:col:15660; institutionCode: NHMUK; collectionCode: Arachnida and Myriapoda; ownerInstitutionCode: NHMUK; basisOfRecord: PreservedSpecimen
- **Type status:** Other material. **Occurrence:** catalogNumber: NHMUK015991351; recordedBy: N. M. Collins; individualCount: 1; sex: male; lifeStage: adult; preparations: preserved in 70% ethanol; occurrenceID: 1F19B0B5-B446-5B2D-B86C-4E0DDC274A78; **Taxon:** scientificName: Mesoschendylajavanica; kingdom: Animalia; phylum: Arthropoda; class: Chilopoda; order: Geophilomorpha; family: Schendylidae; genus: Mesoschendyla; specificEpithet: javanica; taxonRank: species; scientificNameAuthorship: (Attems, 1907); taxonomicStatus: accepted; **Location:** continent: Asia; islandGroup: Greater Sunda Islands; island: Borneo; country: Malaysia; countryCode: MY; stateProvince: Sarawak; locality: Gunung Mulu National Park; verbatimLocality: Gunung Mulu National Park; verbatimElevation: 1310 m; locationRemarks: altitude zonation site G, lower montane forest soil cores; verbatimCoordinates: 4°02'17"N 114°53'10"E; decimalLatitude: 4.038056; decimalLongitude: 114.886111; georeferenceProtocol: label; **Identification:** identifiedBy: George Popovici; dateIdentified: 03-2025; **Event:** samplingProtocol: soil core sample; eventDate: 16/18-02-1978; year: 1978; month: 2; day: 16–18; **Record Level:** collectionID: urn:lsid:biocol.org:col:15660; institutionCode: NHMUK; collectionCode: Arachnida and Myriapoda; ownerInstitutionCode: NHMUK; basisOfRecord: PreservedSpecimen
- **Type status:** Other material. **Occurrence:** catalogNumber: NHMUK015991319; recordedBy: N. M. Collins; individualCount: 1; sex: male; lifeStage: adult; preparations: preserved in 70% ethanol; occurrenceID: 4C3FF14F-12C9-570E-855E-D429EC7EA58E; **Taxon:** scientificName: Mesoschendylajavanica; kingdom: Animalia; phylum: Arthropoda; class: Chilopoda; order: Geophilomorpha; family: Schendylidae; genus: Mesoschendyla; specificEpithet: javanica; taxonRank: species; scientificNameAuthorship: (Attems, 1907); taxonomicStatus: accepted; **Location:** continent: Asia; islandGroup: Greater Sunda Islands; island: Borneo; country: Malaysia; countryCode: MY; stateProvince: Sarawak; locality: Gunung Mulu National Park; verbatimLocality: Gunung Mulu National Park; verbatimElevation: 130 m; locationRemarks: altitude zonation site B, mixed dipterocarp forest soil cores; verbatimCoordinates: 4°03'10"N 114°51'10"E; decimalLatitude: 4.052778; decimalLongitude: 114.852778; georeferenceProtocol: label; **Identification:** identifiedBy: George Popovici; dateIdentified: 03-2025; **Event:** samplingProtocol: soil core sample; eventDate: 31/01-01/02-1978; year: 1978; month: 01–02; day: 31–01; **Record Level:** collectionID: urn:lsid:biocol.org:col:15660; institutionCode: NHMUK; collectionCode: Arachnida and Myriapoda; ownerInstitutionCode: NHMUK; basisOfRecord: PreservedSpecimen
- **Type status:** Other material. **Occurrence:** catalogNumber: NHMUK015991313; recordedBy: N. M. Collins; individualCount: 1; sex: male; lifeStage: adult; preparations: preserved in 70% ethanol; occurrenceID: F05B56CD-500F-52CC-BBCE-1F6FEA1CBAA1; **Taxon:** scientificName: Mesoschendylajavanica; kingdom: Animalia; phylum: Arthropoda; class: Chilopoda; order: Geophilomorpha; family: Schendylidae; genus: Mesoschendyla; specificEpithet: javanica; taxonRank: species; scientificNameAuthorship: (Attems, 1907); taxonomicStatus: accepted; **Location:** continent: Asia; islandGroup: Greater Sunda Islands; island: Borneo; country: Malaysia; countryCode: MY; stateProvince: Sarawak; locality: Gunung Mulu National Park; verbatimLocality: Gunung Mulu National Park; verbatimElevation: 50 m; locationRemarks: altitude zonation site B, mixed dipterocarp forest soil cores; verbatimCoordinates: 4°01'10"N 114°48'22"E; decimalLatitude: 4.019444; decimalLongitude: 114.806111; georeferenceProtocol: label; **Identification:** identifiedBy: George Popovici; dateIdentified: 03-2025; **Event:** samplingProtocol: soil core sample; eventDate: 07/08-02-1978; year: 1978; month: 2; day: 07–08; **Record Level:** collectionID: urn:lsid:biocol.org:col:15660; institutionCode: NHMUK; collectionCode: Arachnida and Myriapoda; ownerInstitutionCode: NHMUK; basisOfRecord: PreservedSpecimen
- **Type status:** Other material. **Occurrence:** catalogNumber: NHMUK015991310; recordedBy: N. M. Collins; individualCount: 1; sex: male; lifeStage: adult; preparations: preserved in 70% ethanol; occurrenceID: B17DACA7-20B5-5F34-B55E-18E9048CDE17; **Taxon:** scientificName: Mesoschendylajavanica; kingdom: Animalia; phylum: Arthropoda; class: Chilopoda; order: Geophilomorpha; family: Schendylidae; genus: Mesoschendyla; specificEpithet: javanica; taxonRank: species; scientificNameAuthorship: (Attems, 1907); taxonomicStatus: accepted; **Location:** continent: Asia; islandGroup: Greater Sunda Islands; island: Borneo; country: Malaysia; countryCode: MY; stateProvince: Sarawak; locality: Gunung Mulu National Park; verbatimLocality: Gunung Mulu National Park; verbatimElevation: 1140 m; locationRemarks: alluvial soil cores; verbatimCoordinates: 4°02'22"N 114°52'58"E; decimalLatitude: 4.039444; decimalLongitude: 114.882778; georeferenceProtocol: label; **Identification:** identifiedBy: George Popovici; dateIdentified: 03-2025; **Event:** samplingProtocol: soil core sample; eventDate: 07/08-02-1978; year: 1978; month: 2; day: 07–08; **Record Level:** collectionID: urn:lsid:biocol.org:col:15660; institutionCode: NHMUK; collectionCode: Arachnida and Myriapoda; ownerInstitutionCode: NHMUK; basisOfRecord: PreservedSpecimen
- **Type status:** Other material. **Occurrence:** catalogNumber: NHMUK015991305; recordedBy: N. M. Collins; individualCount: 5; sex: male; lifeStage: adult; preparations: preserved in 70% ethanol; occurrenceID: ACA74FEE-51FC-59D3-8967-9231546D5F07; **Taxon:** scientificName: Mesoschendylajavanica; kingdom: Animalia; phylum: Arthropoda; class: Chilopoda; order: Geophilomorpha; family: Schendylidae; genus: Mesoschendyla; specificEpithet: javanica; taxonRank: species; scientificNameAuthorship: (Attems, 1907); taxonomicStatus: accepted; **Location:** continent: Asia; islandGroup: Greater Sunda Islands; island: Borneo; country: Malaysia; countryCode: MY; stateProvince: Sarawak; locality: Gunung Mulu National Park; verbatimLocality: Gunung Mulu National Park; verbatimElevation: 2070 m; locationRemarks: altitude zonation site F, lower montane forest soil cores; verbatimCoordinates: 4°02'47"N 114°55'16"E; decimalLatitude: 4.046389; decimalLongitude: 114.921111; georeferenceProtocol: label; **Identification:** identifiedBy: George Popovici; dateIdentified: 03-2025; **Event:** samplingProtocol: soil core sample; eventDate: 16/17-03-1978; year: 1978; month: 3; day: 16–17; **Record Level:** collectionID: urn:lsid:biocol.org:col:15660; institutionCode: NHMUK; collectionCode: Arachnida and Myriapoda; ownerInstitutionCode: NHMUK; basisOfRecord: PreservedSpecimen
- **Type status:** Other material. **Occurrence:** catalogNumber: NHMUK015991302; recordedBy: N. M. Collins; individualCount: 1; sex: male; lifeStage: adult; preparations: preserved in 70% ethanol; occurrenceID: 80091E5A-7F01-5215-8758-9A07D2E7C43E; **Taxon:** scientificName: Mesoschendylajavanica; kingdom: Animalia; phylum: Arthropoda; class: Chilopoda; order: Geophilomorpha; family: Schendylidae; genus: Mesoschendyla; specificEpithet: javanica; taxonRank: species; scientificNameAuthorship: (Attems, 1907); taxonomicStatus: accepted; **Location:** continent: Asia; islandGroup: Greater Sunda Islands; island: Borneo; country: Malaysia; countryCode: MY; stateProvince: Sarawak; locality: Gunung Mulu National Park; verbatimLocality: Gunung Mulu National Park; verbatimElevation: 1860 m; locationRemarks: altitude zonation site K, upper montane forest soil cores; verbatimCoordinates: 4°02'44"N 114°54'59"E; decimalLatitude: 4.045556; decimalLongitude: 114.916389; georeferenceProtocol: label; **Identification:** identifiedBy: George Popovici; dateIdentified: 03-2025; **Event:** samplingProtocol: soil core sample; eventDate: 21-02-1978; year: 1978; month: 2; day: 21; **Record Level:** collectionID: urn:lsid:biocol.org:col:15660; institutionCode: NHMUK; collectionCode: Arachnida and Myriapoda; ownerInstitutionCode: NHMUK; basisOfRecord: PreservedSpecimen
- **Type status:** Other material. **Occurrence:** catalogNumber: NHMUK015991298; recordedBy: N. M. Collins; individualCount: 2; sex: male; lifeStage: adult; preparations: preserved in 70% ethanol; occurrenceID: 69DA3646-386C-53FC-8BD4-DBBAC6F8F367; **Taxon:** scientificName: Mesoschendylajavanica; kingdom: Animalia; phylum: Arthropoda; class: Chilopoda; order: Geophilomorpha; family: Schendylidae; genus: Mesoschendyla; specificEpithet: javanica; taxonRank: species; scientificNameAuthorship: (Attems, 1907); taxonomicStatus: accepted; **Location:** continent: Asia; islandGroup: Greater Sunda Islands; island: Borneo; country: Malaysia; countryCode: MY; stateProvince: Sarawak; locality: Gunung Mulu National Park; verbatimLocality: Gunung Mulu National Park; verbatimElevation: 200–250 m; locationRemarks: mixed dipterocarp forest soil cores; verbatimCoordinates: 4°03'00"N 114°51'40"E; decimalLatitude: 4.05; decimalLongitude: 114.861111; georeferenceProtocol: label; **Identification:** identifiedBy: George Popovici; dateIdentified: 03-2025; **Event:** samplingProtocol: soil core sample; eventDate: 02-06-1978; year: 1978; month: 6; day: 2; **Record Level:** collectionID: urn:lsid:biocol.org:col:15660; institutionCode: NHMUK; collectionCode: Arachnida and Myriapoda; ownerInstitutionCode: NHMUK; basisOfRecord: PreservedSpecimen
- **Type status:** Other material. **Occurrence:** catalogNumber: NHMUK015991298; recordedBy: N. M. Collins; individualCount: 1; sex: female; lifeStage: adult; preparations: preserved in 70% ethanol; occurrenceID: BCFCA42D-B4A1-5728-BBFD-76DC4D6D48C9; **Taxon:** scientificName: Mesoschendylajavanica; kingdom: Animalia; phylum: Arthropoda; class: Chilopoda; order: Geophilomorpha; family: Schendylidae; genus: Mesoschendyla; specificEpithet: javanica; taxonRank: species; scientificNameAuthorship: (Attems, 1907); taxonomicStatus: accepted; **Location:** continent: Asia; islandGroup: Greater Sunda Islands; island: Borneo; country: Malaysia; countryCode: MY; stateProvince: Sarawak; locality: Gunung Mulu National Park; verbatimLocality: Gunung Mulu National Park; verbatimElevation: 200–250 m; locationRemarks: mixed dipterocarp forest soil cores; verbatimCoordinates: 4°03'00"N 114°51'40"E; decimalLatitude: 4.05; decimalLongitude: 114.861111; georeferenceProtocol: label; **Identification:** identifiedBy: George Popovici; dateIdentified: 03-2025; **Event:** samplingProtocol: soil core sample; eventDate: 02-06-1978; year: 1978; month: 6; day: 2; **Record Level:** collectionID: urn:lsid:biocol.org:col:15660; institutionCode: NHMUK; collectionCode: Arachnida and Myriapoda; ownerInstitutionCode: NHMUK; basisOfRecord: PreservedSpecimen
- **Type status:** Other material. **Occurrence:** catalogNumber: NHMUK015991294; recordedBy: N. M. Collins; individualCount: 2; sex: male; lifeStage: adult; preparations: preserved in 70% ethanol; occurrenceID: 9BD61FD9-63D9-5A62-B58B-083C49934C3C; **Taxon:** scientificName: Mesoschendylajavanica; kingdom: Animalia; phylum: Arthropoda; class: Chilopoda; order: Geophilomorpha; family: Schendylidae; genus: Mesoschendyla; specificEpithet: javanica; taxonRank: species; scientificNameAuthorship: (Attems, 1907); taxonomicStatus: accepted; **Location:** continent: Asia; islandGroup: Greater Sunda Islands; island: Borneo; country: Malaysia; countryCode: MY; stateProvince: Sarawak; locality: Gunung Mulu National Park; verbatimLocality: Gunung Mulu National Park; verbatimElevation: 200–250 m; locationRemarks: mixed dipterocarp forest soil cores; verbatimCoordinates: 4°03'00"N 114°51'40"E; decimalLatitude: 4.05; decimalLongitude: 114.861111; georeferenceProtocol: label; **Identification:** identifiedBy: George Popovici; dateIdentified: 03-2025; **Event:** samplingProtocol: soil core sample; eventDate: 13/14-02-1978; year: 1978; month: 2; day: 13–14; **Record Level:** collectionID: urn:lsid:biocol.org:col:15660; institutionCode: NHMUK; collectionCode: Arachnida and Myriapoda; ownerInstitutionCode: NHMUK; basisOfRecord: PreservedSpecimen
- **Type status:** Other material. **Occurrence:** catalogNumber: NHMUK015991294; recordedBy: N. M. Collins; individualCount: 1; sex: female; lifeStage: adult; preparations: preserved in 70% ethanol; occurrenceID: CCEB2260-4CDF-573B-8724-47149F54FA08; **Taxon:** scientificName: *Mesoschendylajavanica*; kingdom: Animalia; phylum: Arthropoda; class: Chilopoda; order: Geophilomorpha; family: Schendylidae; genus: Mesoschendyla; specificEpithet: *javanica*; taxonRank: species; scientificNameAuthorship: (Attems, 1907); taxonomicStatus: accepted; **Location:** continent: Asia; islandGroup: Greater Sunda Islands; island: Borneo; country: Malaysia; countryCode: MY; stateProvince: Sarawak; locality: Gunung Mulu National Park; verbatimLocality: Gunung Mulu National Park; verbatimElevation: 200–250 m; locationRemarks: mixed dipterocarp forest soil cores; verbatimCoordinates: 4°03'00"N 114°51'40"E; decimalLatitude: 4.05; decimalLongitude: 114.861111; georeferenceProtocol: label; **Identification:** identifiedBy: George Popovici; dateIdentified: 03-2025; **Event:** samplingProtocol: soil core sample; eventDate: 13/14-02-1978; year: 1978; month: 2; day: 13–14; **Record Level:** collectionID: urn:lsid:biocol.org:col:15660; institutionCode: NHMUK; collectionCode: Arachnida and Myriapoda; ownerInstitutionCode: NHMUK; basisOfRecord: PreservedSpecimen
- **Type status:** Other material. **Occurrence:** catalogNumber: NHMUK015991293; recordedBy: N. M. Collins; individualCount: 8; sex: male; lifeStage: adult; preparations: preserved in 70% ethanol; occurrenceID: FFABA21F-A8D7-5673-B174-3DA7822E175A; **Taxon:** scientificName: Mesoschendylajavanica; kingdom: Animalia; phylum: Arthropoda; class: Chilopoda; order: Geophilomorpha; family: Schendylidae; genus: Mesoschendyla; specificEpithet: javanica; taxonRank: species; scientificNameAuthorship: (Attems, 1907); taxonomicStatus: accepted; **Location:** continent: Asia; islandGroup: Greater Sunda Islands; island: Borneo; country: Malaysia; countryCode: MY; stateProvince: Sarawak; locality: Gunung Mulu National Park; verbatimLocality: Gunung Mulu National Park; verbatimElevation: 550 m; locationRemarks: altitude zonation site D, mixed dipterocarp forest soil cores; verbatimCoordinates: 4°02'32"N 114°52'14"E; decimalLatitude: 4.042222; decimalLongitude: 114.870556; georeferenceProtocol: label; **Identification:** identifiedBy: George Popovici; dateIdentified: 03-2025; **Event:** samplingProtocol: soil core sample; eventDate: 02/05-02-1978; year: 1978; month: 2; day: 02–05; **Record Level:** collectionID: urn:lsid:biocol.org:col:15660; institutionCode: NHMUK; collectionCode: Arachnida and Myriapoda; ownerInstitutionCode: NHMUK; basisOfRecord: PreservedSpecimen
- **Type status:** Other material. **Occurrence:** catalogNumber: NHMUK015991267; recordedBy: N. M. Collins; individualCount: 6; sex: male; lifeStage: adult; preparations: preserved in 70% ethanol; occurrenceID: 34950A9F-D14D-521F-A925-59CC5D2D46AE; **Taxon:** scientificName: Mesoschendylajavanica; kingdom: Animalia; phylum: Arthropoda; class: Chilopoda; order: Geophilomorpha; family: Schendylidae; genus: Mesoschendyla; specificEpithet: javanica; taxonRank: species; scientificNameAuthorship: (Attems, 1907); taxonomicStatus: accepted; **Location:** continent: Asia; islandGroup: Greater Sunda Islands; island: Borneo; country: Malaysia; countryCode: MY; stateProvince: Sarawak; locality: Gunung Mulu National Park; verbatimLocality: Gunung Mulu National Park; verbatimElevation: 200–250 m; locationRemarks: mixed dipterocarp forest soil cores; verbatimCoordinates: 4°03'00"N 114°51'40"E; decimalLatitude: 4.05; decimalLongitude: 114.861111; georeferenceProtocol: label; **Identification:** identifiedBy: George Popovici; dateIdentified: 03-2025; **Event:** samplingProtocol: soil core sample; eventDate: 03/04-02-1978; year: 1978; month: 2; day: 03–04; **Record Level:** collectionID: urn:lsid:biocol.org:col:15660; institutionCode: NHMUK; collectionCode: Arachnida and Myriapoda; ownerInstitutionCode: NHMUK; basisOfRecord: PreservedSpecimen
- **Type status:** Other material. **Occurrence:** catalogNumber: NHMUK015991266; recordedBy: N. M. Collins; individualCount: 1; sex: male; lifeStage: adult; preparations: preserved in 70% ethanol; occurrenceID: E4A0A3BA-E5C8-5A91-8D4A-D4B057305981; **Taxon:** scientificName: Mesoschendylajavanica; kingdom: Animalia; phylum: Arthropoda; class: Chilopoda; order: Geophilomorpha; family: Schendylidae; genus: Mesoschendyla; specificEpithet: javanica; taxonRank: species; scientificNameAuthorship: (Attems, 1907); taxonomicStatus: accepted; **Location:** continent: Asia; islandGroup: Greater Sunda Islands; island: Borneo; country: Malaysia; countryCode: MY; stateProvince: Sarawak; locality: Gunung Mulu National Park; verbatimLocality: Gunung Mulu National Park; verbatimElevation: 1310 m; locationRemarks: altitude zonation site G, lower montane forest soil cores; verbatimCoordinates: 4°02'17"N 114°53'10"E; decimalLatitude: 4.038056; decimalLongitude: 114.886111; georeferenceProtocol: label; **Identification:** identifiedBy: George Popovici; dateIdentified: 03-2025; **Event:** samplingProtocol: soil core sample; eventDate: 16/18-02-1978; year: 1978; month: 2; day: 16–18; **Record Level:** collectionID: urn:lsid:biocol.org:col:15660; institutionCode: NHMUK; collectionCode: Arachnida and Myriapoda; ownerInstitutionCode: NHMUK; basisOfRecord: PreservedSpecimen
- **Type status:** Other material. **Occurrence:** catalogNumber: NHMUK015991257; recordedBy: N. M. Collins; individualCount: 1; sex: male; lifeStage: adult; preparations: preserved in 70% ethanol; occurrenceID: 69DBFC14-566C-5B8C-9D59-07DA18ACA69F; **Taxon:** scientificName: Mesoschendylajavanica; kingdom: Animalia; phylum: Arthropoda; class: Chilopoda; order: Geophilomorpha; family: Schendylidae; genus: Mesoschendyla; specificEpithet: javanica; taxonRank: species; scientificNameAuthorship: (Attems, 1907); taxonomicStatus: accepted; **Location:** continent: Asia; islandGroup: Greater Sunda Islands; island: Borneo; country: Malaysia; countryCode: MY; stateProvince: Sarawak; locality: Gunung Mulu National Park; verbatimLocality: Gunung Mulu National Park; verbatimElevation: 500 m; locationRemarks: humus, mixed dipterocarp forest near Camp 2; verbatimCoordinates: 4°02'38"N 114°52'13"E; decimalLatitude: 4.043889; decimalLongitude: 114.870278; georeferenceProtocol: label; **Identification:** identifiedBy: George Popovici; dateIdentified: 03-2025; **Event:** samplingProtocol: soil core sample; eventDate: 01-08-1978; year: 1978; month: 8; day: 1; fieldNumber: C52; **Record Level:** collectionID: urn:lsid:biocol.org:col:15660; institutionCode: NHMUK; collectionCode: Arachnida and Myriapoda; ownerInstitutionCode: NHMUK; basisOfRecord: PreservedSpecimen
- **Type status:** Syntype. **Occurrence:** catalogNumber: NHMW-MY4883; recordedBy: K. Kraepelin; individualCount: 1; lifeStage: adult; preparations: slide-mounted; occurrenceID: 070EC925-F8C0-5EC7-B016-CD0725159314; **Taxon:** scientificName: *Mesoschendylajavanica*; kingdom: Animalia; phylum: Arthropoda; class: Chilopoda; order: Geophilomorpha; family: Schendylidae; genus: Mesoschendyla; specificEpithet: *javanica*; taxonRank: species; scientificNameAuthorship: (Attems, 1907); taxonomicStatus: accepted; **Location:** continent: Asia; islandGroup: Greater Sunda Islands; island: Java; country: Indonesia; countryCode: ID; stateProvince: West Java; municipality: Jakarta Raya; locality: Ciampea; verbatimLocality: Tjompea; georeferenceProtocol: label; **Identification:** identifiedBy: George Popovici; dateIdentified: 03-2025; **Event:** samplingProtocol: sieved from bat guano; eventDate: 1903; year: 1903; **Record Level:** collectionID: urn:lsid:biocol.org:col:15588; institutionCode: NHMW; collectionCode: Myriapoda; ownerInstitutionCode: NHMW; basisOfRecord: PreservedSpecimen
- **Type status:** Syntype. **Occurrence:** catalogNumber: NHMW-MY10655; recordedBy: K. Kraepelin; individualCount: 1; sex: male; lifeStage: adult; preparations: slide-mounted; occurrenceID: D31B4119-5F97-53AE-B955-86AB51DABA70; **Taxon:** scientificName: *Mesoschendylajavanica*; kingdom: Animalia; phylum: Arthropoda; class: Chilopoda; order: Geophilomorpha; family: Schendylidae; genus: Mesoschendyla; specificEpithet: *javanica*; taxonRank: species; scientificNameAuthorship: (Attems, 1907); taxonomicStatus: accepted; **Location:** continent: Asia; islandGroup: Greater Sunda Islands; island: Java; country: Indonesia; countryCode: ID; stateProvince: West Java; municipality: Jakarta Raya; locality: Ciampea; verbatimLocality: Tjompea; georeferenceProtocol: label; **Identification:** identifiedBy: George Popovici; dateIdentified: 03-2025; **Event:** samplingProtocol: sieved from bat guano; eventDate: 1903; year: 1903; **Record Level:** collectionID: urn:lsid:biocol.org:col:15588; institutionCode: NHMW; collectionCode: Myriapoda; ownerInstitutionCode: NHMW; basisOfRecord: PreservedSpecimen

#### Description

**Habitus**. Body length 7–8.5 mm [10 mm, including antennae]. Most specimens with 31 leg-bearing segments, one female (NHMUK015991298) with 33. Colour in ethanol white.

**Figure 1.**
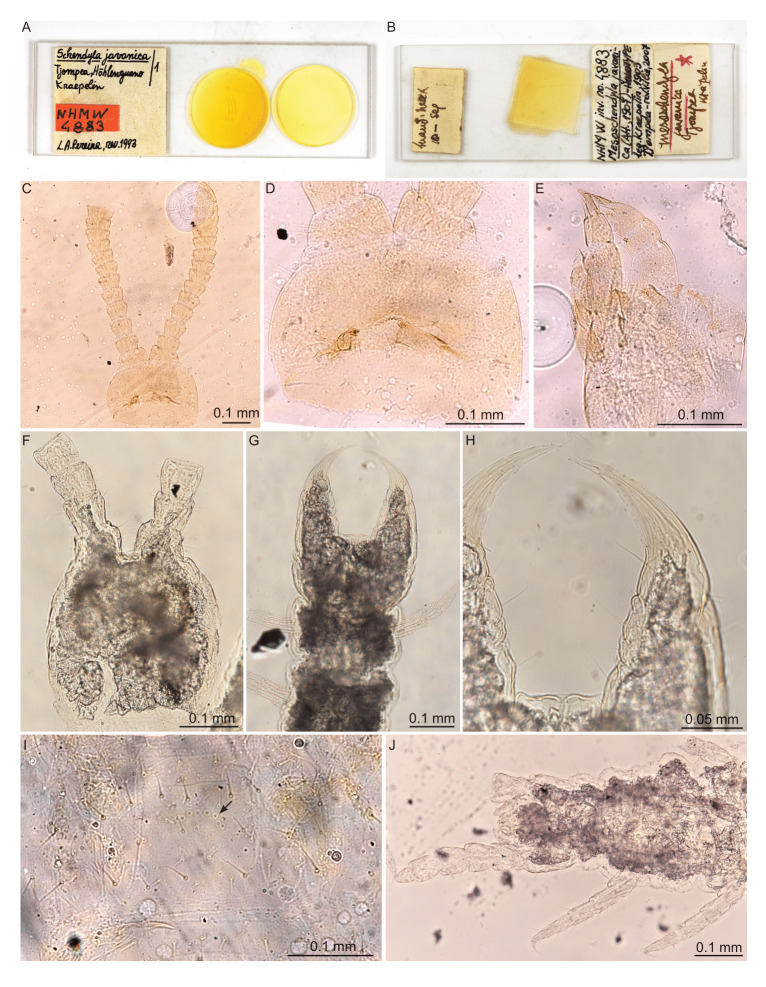
*Mesoschendylajavanica* (Attems, 1907). **A**, **B** Overview of slide-mounted syntypes in the NHMW collection; **C** NHMW-MY4883, cephalic capsule and antennae (ventral view); **D** NHMW-MY4883, clypeus and labrum with mandibles in situ (ventral view); **E** NHMW-MY4883, maxillae (lateral view); **F** NHMW-MY10655, cephalic capsule and antennae (dorsal view); **G** NHMW-MY10655, forcipular segment and anterior part of body (ventral view); **H** NHMW-MY10655, forcipules (ventral view); **I** NHMW-MY10655, anterior metasternite showing pore field (indicated by black arrow) (ventral view); **J** NHMW-MY10655, posterior part of body (ventral view).

**Figure 2.**
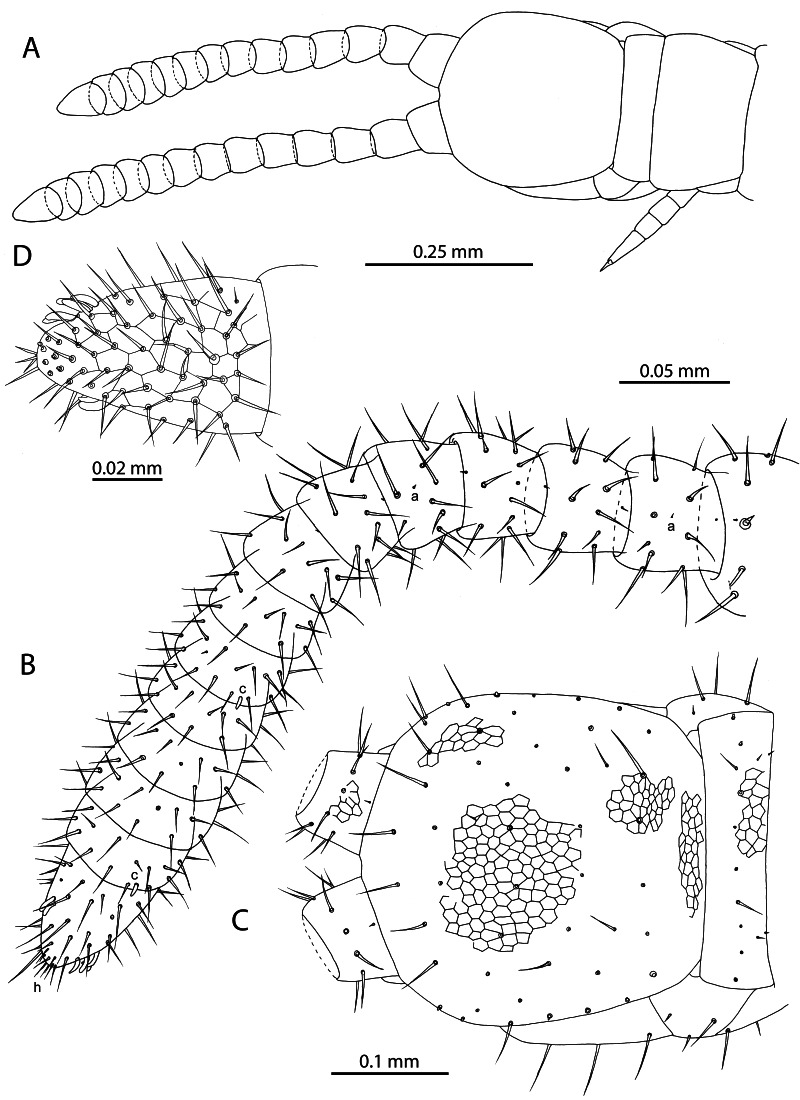
*Mesoschendylajavanica*. **A** NHMUK015991355, head and anterior end of trunk (dorsal view); **B** NHMUK015991354, left antenna with specialised sensilla indicated (dorsal view); **C** NHMUK015991355, cephalic capsule (dorsal view); **D** NHMUK015991403, antennal article XIV (ventral view). a = sensillum microtrichodeum, c = sensillum basiconicum, h = undivided hyaline sensilla.

**Head and antennae** (Fig. 1C, F; Fig. 2). Antennae 0.5–0.6 mm long, 2.4 times longer than cephalic capsule fully extended (Fig. 2A) [Fig. 1C]. Articles II and V with specialised sensillum microtrichodeum (type a sensu Pereira et al. (2000)) localised medially on the dorsal side (Fig. 2B). Articles IX and XIII with specialised sensillum basiconicum (type c sensu Pereira et al. (2000)) localised at distal edge on dorsal side (Fig. 2B). Starting from article VI, the distal antennal articles become moderately enlarged resulting in a mildly clavate general appearance (Fig. 2B) [Fig. 1C]. In ethanol, most specimens show weak geniculation of the antenna at article VI (Fig. 2B). Article XIV 1.5 times longer than wide with two groups of sensilla basiconica laterally and an apical group of small undivided hyaline sensilla (Fig. 2D). Cephalic plate nearly pentagonal, weakly narrowed posteriorly, about as long as broad, with generally uniform reticulation on dorsal side. Chaetotaxy as in Fig. 2C.

**Figure 3.**
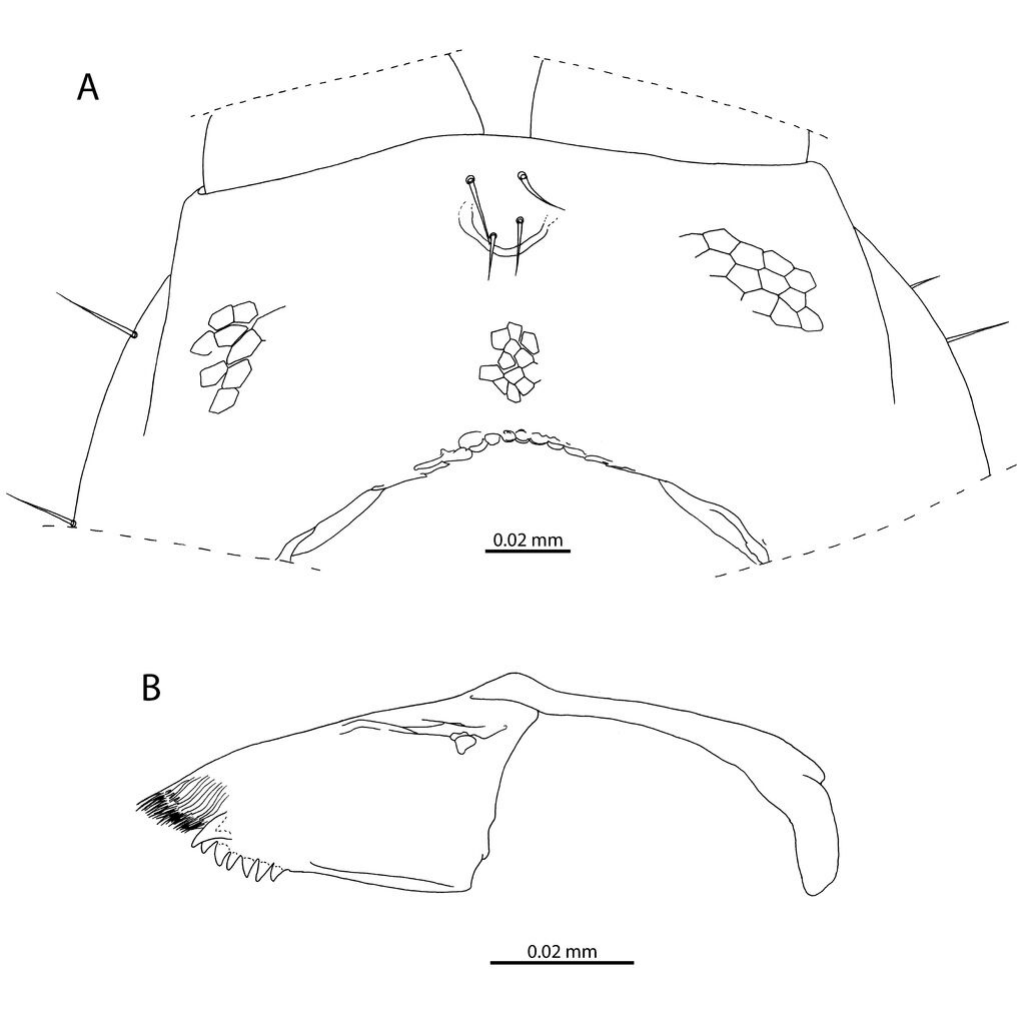
*Mesoschendylajavanica*. **A** NHMUK015991403, clypeus and labrum (ventral view); **B** NHMUK015991403, right mandible (dorsal view).

**Clypeus** (Fig. 1D; Fig. 3A). Inconspicuous reticulation across entire surface. One pair of postantennal and one pair of medial sensilla. Clypeal area and prelabral setae absent.

**Labrum** (Fig. 1D; Fig. 3A). Gently curved, bearing 9–12 tubercles [9 tubercles, Fig. 1D]. Medial tubercles with sclerotised rim apically. Lateral tubercles minute, medially recurved and apically tapering.

**Mandible** (Fig. 1D; Fig. 3B). With one single pectinate lamella and one dentate lamella. Dentate lamella bearing seven subequal curved tooth-like projections [six visible, Fig. 1D]. Projections of dentate lamella forming single contiguous row, not partitioned into blocks.

**Figure 4.**
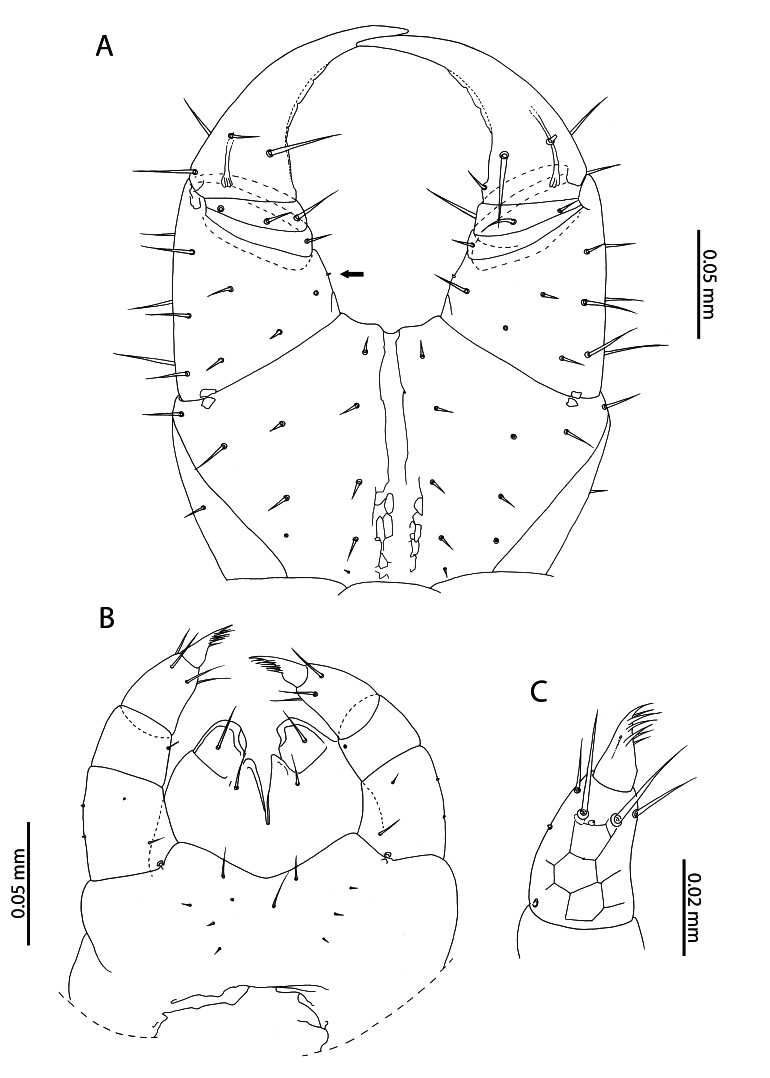
*Mesoschendylajavanica*. **A** NHMUK015991403, forcipular segment, sensillum coeloconicum indicated by black arrow (ventral view); **B** NHMUK015991403, maxillae (ventral view); **C** NHMUK015991403, article 3 and pretarsus of left second maxillary telopodite (ventral view).

**Maxillae** (Fig. 1E; Fig. 4B, C). First maxillae without telopodital or coxosternal lappets. First maxillae with triangular coxosternal projections, each bearing one seta and uni-articulate telopodites, with one seta each (Fig. 4B). Second maxillary coxosternite with rounded, concave anterior margin. Telopodite short, robust, ending in claw-shaped pretarsus with pectinate lateral edges bearing gracile hyaline projections (Fig. 4C) [Fig. 1E]. Chaetotaxy as in Fig. 4B.

**Forcipular segment** (Fig. 1G, H; Fig. 4A). Forcipular coxosternite 1.7 times broader than long (Fig. 4A) [Fig. 1G]. Chitin lines absent. Anterior margin broadly concave, lacking denticles or other projections. Forcipular trochanteroprefemur 1.1 times longer than broad, without denticles, but bearing one minute sensillum coeloconicum on internal side. Tarsungulum with basal bulge, but lacking basal denticle. Inner concavity with three broad, rectangular serrations. Calyx of venom gland visible in base of tarsungulum, ovoid. Chaetotaxy as in Fig. 4A.

**Figure 5.**
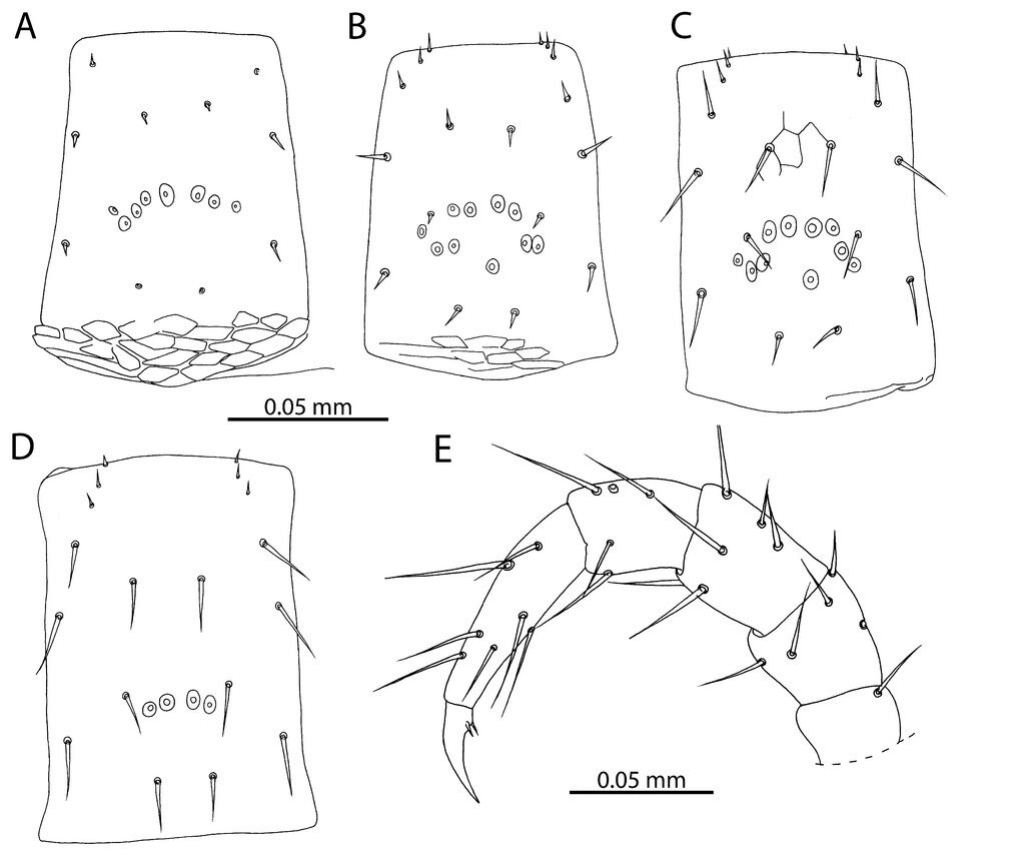
*Mesoschendylajavanica*. **A** NHMUK015991403, metasternite 2 (ventral view); **B** NHMUK015991403, metasternite 5 (ventral view); **C** NHMUK015991403, metasternite 8 (ventral view); **D** NHMUK015991403, metasternite 12 (ventral view); **E** NHMUK015991354, leg 30 (lateral view).

**Trunk** (Fig. 1I; Fig. 5A–D). Anterior metasternites 2–12 with pore field comprising 4–10 pores localised postero-medially (Fig. 5A–D) [Fig. 1I]. No bulges or changes in cuticular structure observed around pore fields. Shape of pore fields anteriorly a posteriorly curved line, becoming an interrupted ovoid on metasternites 4–10 and transitioning again to a posteriorly curved line on metasternites 11–12. Chaetotaxy illustrated in Fig. 5A–D, comprising two internal rows of three macrosetae, two external rows of three macrosetae and two anteriorly recurved rows of microsetae at anterior margin. Walking legs with uniform chaetotaxy (Fig. 5E). Claw-shaped pretarsus with two basal accessory spines on the ventral side.

**Figure 6.**
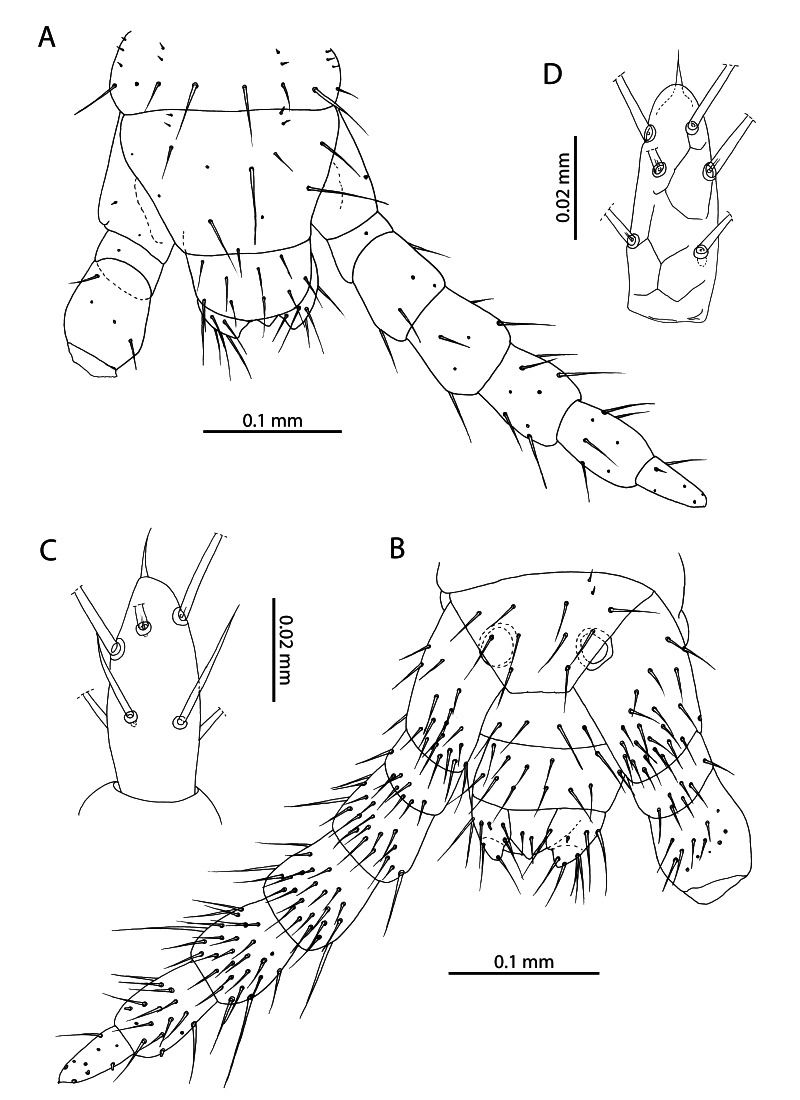
*Mesoschendylajavanica*. **A** NHMUK015991355, male ultimate leg-bearing segment (dorsal view); **B** NHMUK015991355, male ultimate leg-bearing segment (ventral view); **C**, **D** NHMUK015991354, ultimate leg metatarsus of male (lateral views).

**Figure 7.**
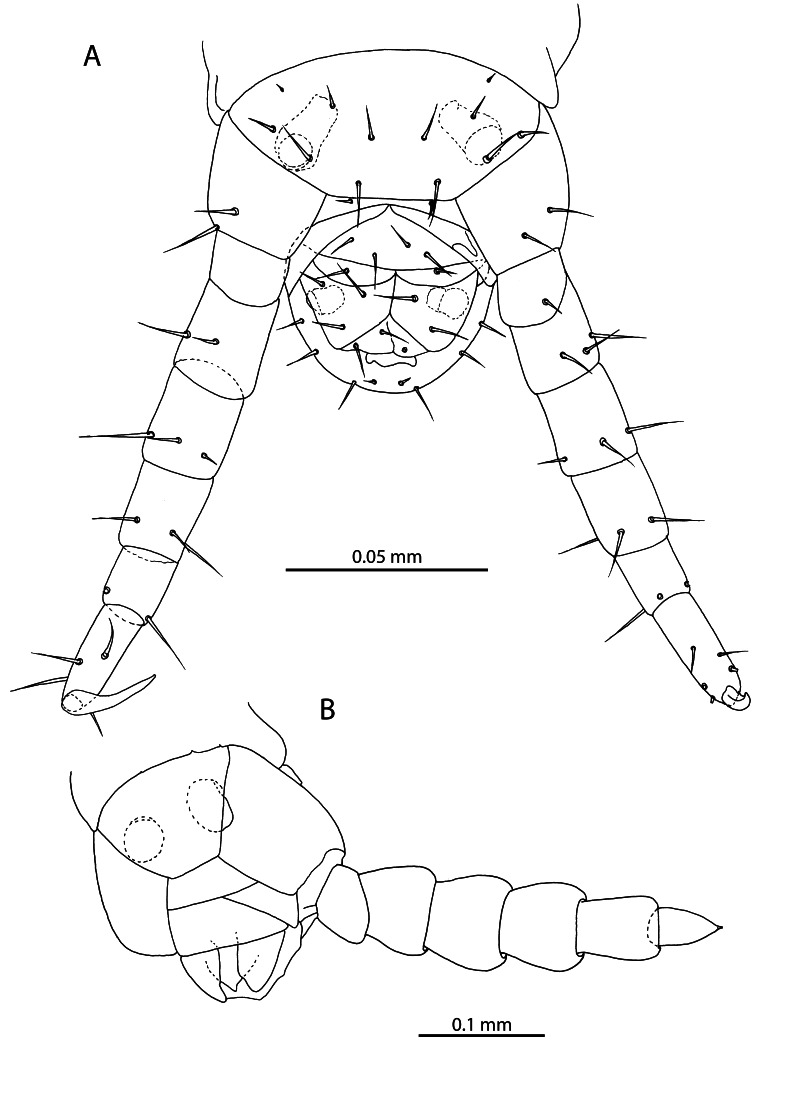
*Mesoschendylajavanica*. **A** NHMUK015991354, female ultimate leg-bearing segment (ventral view); **B** NHMUK015991352, male ultimate leg-bearing segment, setae not illustrated (ventral view).

**Figure 8.**
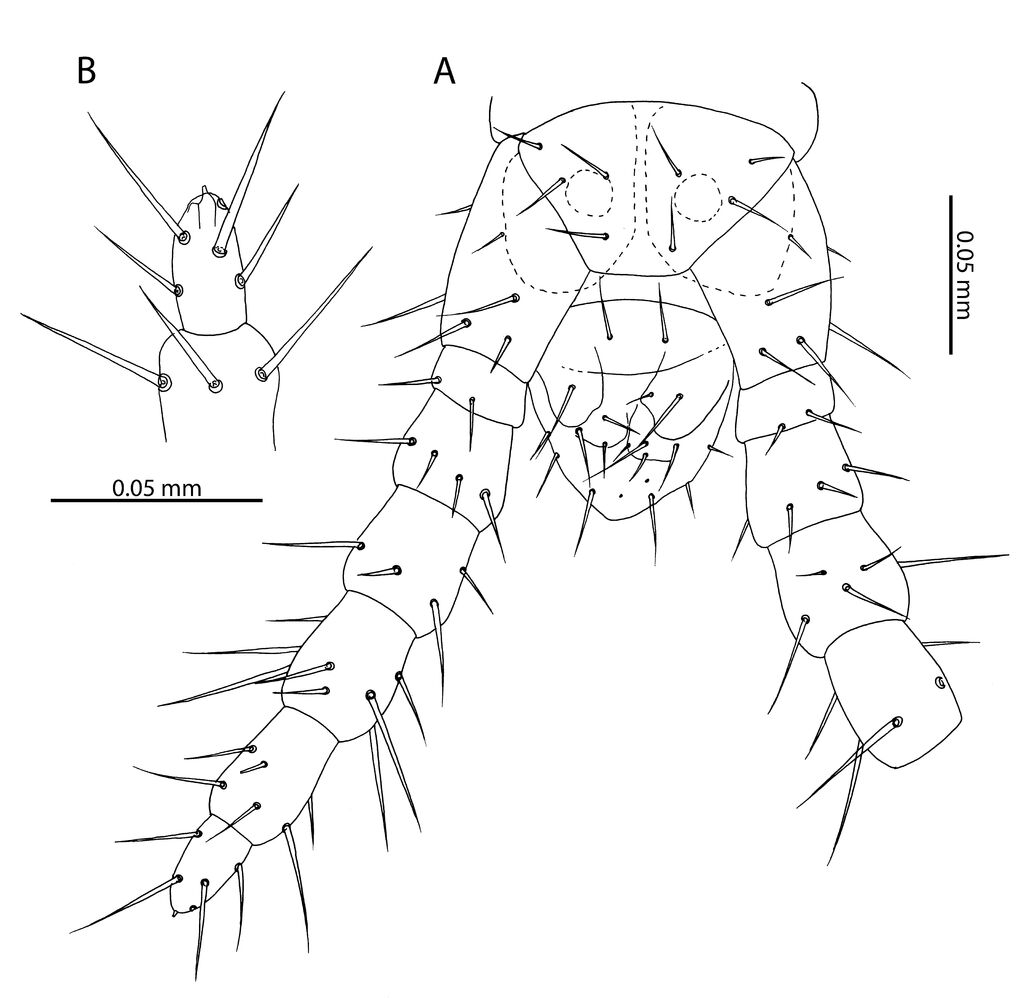
*Mesoschendylajavanica*. **A** NHMUK015991403, male ultimate leg-bearing segment (ventral view); **B** NHMUK015991403, ultimate leg metatarsus of male (ventral view).

**Last leg-bearing segment** (Fig. 1J; Figs 6, 7, 8). Ultimate pleuropretergite whole (Fig. 6A). Ultimate metatergite trapezoidal, with straight posterior edge, 1.5x broader than long. Ultimate metasternite trapezoidal, 1.6–1.7 times broader than long in male (Fig. 6B) and 2x broader than long in female (Fig. 7A). Coxopleura moderately enlarged, with glandular mass opening in a single coxal organ, usually entirely covered by metasternite. In adult males, distal end of coxopleuron covered by dense field of setae (Fig. 6B). Ultimate leg telopodite of mature males densely setose ventrally and sparsely setose dorsally, with strongly enlarged trochanter, prefemur, femur and tibia and moderately enlarged tarsus and metatarsus; coxopleuron and telopodite more sparsely setose in small males (Fig. 8A). Metatarsus ending in minute hair-like projection in male (Fig. 6C–D) (frequently broken as in Fig. 8B); large, claw-shaped pretarsus in female (Fig. 7A). Male gonopods weakly biarticulate (articulation frequently inconspicuous), with distinct internal notch, flanking conical penis (Fig. 6B). Female gonopods uni-articulate, short, gently rounded and bearing one pair of setae (Fig. 7A). Anal pores present in both sexes, but frequently covered by genital segments in males.

#### Diagnosis

Body length 7–8.5 mm. 31–33 leg-bearing segments. Antennae weakly clavate and geniculate starting from article VI. Clypeus with one pair of postantennal and one pair of medial setae. Labrum with 9–12 tubercles. First maxillae lacking telopodital or coxosternal lappets. Tarsungulum with three broad, rectangular serrations along its inner concavity. Trunk metasternites 2–12 with pore field transitioning from single posteriorly curved row of pores to open ellipsoid shape and back to single posteriorly curved row of pores. Female ultimate leg telopodite with large claw-shaped pretarsus. Ultimate leg telopodite strongly sexually dimorphic; inflated and lacking pretarsus in male.

#### Distribution

**Figure 9.**
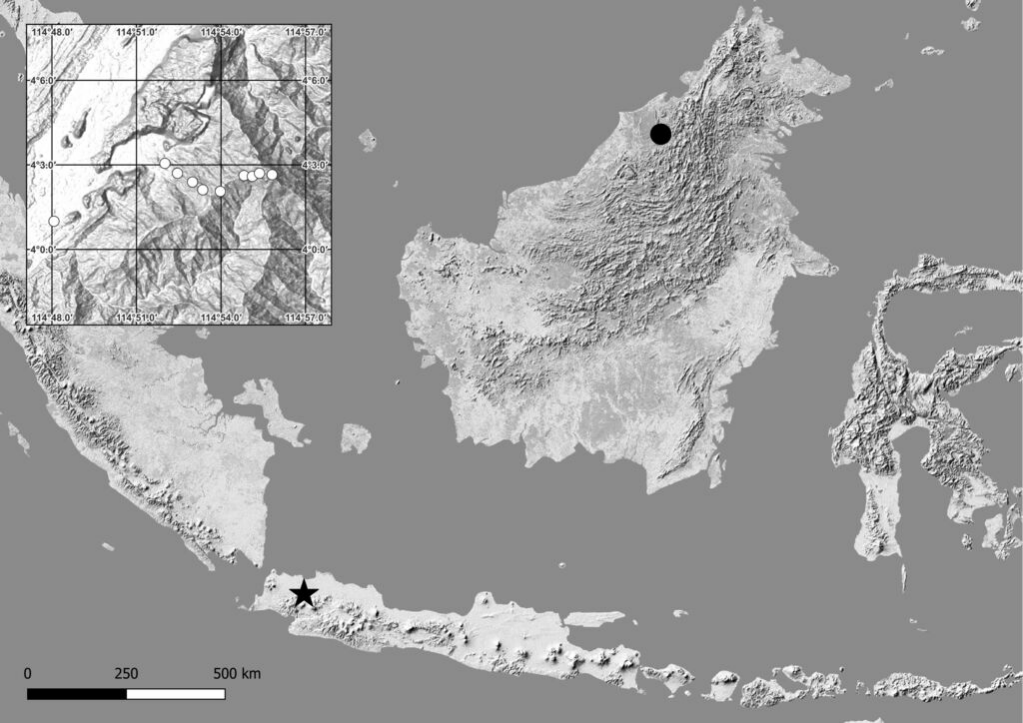
Geographical distribution of known records of *Mesoschendylajavanica* (Attems, 1907). Panel near top left corner showing enlarged overview of Gunung Mulu National Park, where all new specimens presented here have been collected. black star = type locality, black circle = location of Gunung Mulu National Park in northern Borneo, white circle = georeferenced location from which *M.javanica* has been sampled.

West Java (Bogor Regency, Indonesia) and northern Borneo (Sarawak, Malaysia), Greater Sunda Islands (Fig. 9).

#### Ecology

The majority of specimens in the present sample were obtained from 10 cm diameter soil cores at depth of 10–20 cm manually searched for arthropods (Collins 1980), with only one (NHMUK015991403) being hand collected.

Abundance of centipedes in the soil core samples examined by Collins (1980) did not show any clear trend across the altitudinal transect sampled, with the majority of specimens being found in altitudinal zones B (52), J (52), K (57) and L (41). However, as a percentage of total macrofauna sampled, centipedes were recorded as more dominant at the higher altitude zonation sites J (9%), K (11%) and L (28%), only comprising 1–4% at the remaining sites (Collins 1980). Most *M.javanica* specimens (53%) have been collected from soil cores of mixed dipterocarp forest sites at altitudes below 500 m. Specimens with clear altitude zonation site data revealed that of the total number of centipedes sampled at each site (Collins 1980), *M.javanica* specimens accounted for 1.9% at site B (130 m), 34.4% at site D (500 m), 3.1% at site F (1130 m), 7.1% at site G (1310 m), 2.7% at site H (1650 m), 4.6% at site I (1860 m), 13.5% at site J (1930 m), 8.7% at site K (2070 m) and 2.4% at site L (2376 m).

#### Taxon discussion

The species diagnosis above identifies characters shared by the Java and Sarawak material that are consistent with their conspecificity. These include body size, an overlapping number of leg-bearing segments (overwhelmingly 31), restriction of pore fields to sternites 2–11 or 12 and the shape of the pore fields, lack of lappets on the first maxilla and clavate antennae.

The syntype male preserving the ultimate legs shows them to have a slightly shorter metatarsus than most Borneo specimens (Fig. 1J), but proportions of the tarsus change markedly through ontogeny and some large specimens from Borneo (Fig. 7B) match the syntype with respect to proportions of the podomeres.

*Mesoschendylajavanica* exhibits a relatively large degree of sexual dimorphism for Schendylidae. Other genera with the ultimate leg pair of males likewise swollen include *Escaryus* Cook and Collins, 1890 (Cook and Collins 1890), *Plesioschendyla* Ribaut, 1923 (Ribaut 1923), *Marsikomerus* Attems, 1938 (Attems 1938) and *Gosendyla* Chamberlin, 1960 (Chamberlin 1960). Presence or absence of a pretarsal claw on the ultimate leg is usually fixed in schendylid genera and the sexual dimorphism of this trait within a species (present in females, absent in males of *M.javanica*) is exceptional. Remarkably, all juvenile specimens in the present sample appear to be male according to the morphology of the ultimate leg telopodite (Fig. 8A). As only seven of 49 examined specimens are female, it is possible that females in earlier developmental stages are absent from the present samples.

## Discussion

This study provides another example illustrating the key role of the natural history collections in taxonomic research and answering biodiversity related questions. The species *Mesoschendylajavanica* is here studied 122 years after its original discovery in the same geographic area and is redescribed from a larger collection sample that allows us to include important information on its morphology, distribution and habitat preferences. The latter collection, from a Borneo expedition nearly 50 years ago and taxonomically determined for the first time now, provides historic baseline data on Sarawak. The type specimens originally studied by Attems (1907) were, as mentioned earlier, partly lost, except for two micro-preparations kept in the Natural History Museum Vienna (Thofern et al. 2021). Though damaged, these withstood the course of time and delivered helpful information to confirm the species determination. The examination and documentation of these type specimens using modern microscopy further revealed diagnostic morphological characters for the species, which we illustrated to enhance its original description (Attems 1907). Although the type material contained in the micropreparations consists of more than one specimen, we have elected not to designate a lectotype as it remained impossible for us to determine with confidence which of the two was used for the original description of the species (although one of the two slides was originally prepared by Attems himself) and the fact that our redescription relies on the examination of structures from both microslides.

New distribution data for *M.javanica* and noted differences in the biotope of its two localities in Java and Borneo, respectively suggest a wider distribution across the Greater Sunda Islands is very likely for this taxon. Given its presence in soil from multiple forest types (Anderson et al. 1983, Proctor et al. 1983) and across a wide altitudinal range (Collins 1980), we infer that appropriate sampling will likely uncover more specimens from this bioregion.

### Sampling considerations for collecting small-sized Geophilomorpha

The effectiveness of different sampling strategies for quantifying centipede diversity at a given study site has been extensively evaluated in temperate forest, disturbed plain and riverside habitats in Europe (David et al. 1999, Ruiz and Serra 2003, Tuf 2015, Leśniewska and Leśniewski 2016, Baba et al. 2023), but limited comparative data are available from tropical habitats (Druce et al. 2004), including rainforests (Oliveira et al. 2019, Tulande-M et al. 2020). The present material is almost completely sourced from manual soil core sorting, with the other main collection technique employed in sampling centipedes during the RGS expedition being hand collecting from the upper soil layers and soil annexes, yielding only one of 49 examined specimens of *M.javanica*. Amongst ecological groups of centipedes defined by sampling strategy efficiency in Czech floodplain forests (Tuf 2015), *M.javanica* appears to belong to the “Scarcer geophilomorphs” group, which includes taxa only effectively sampled by soil core extraction.

Relative abundance and species diversity of Geophilomorpha at a given sampling site remain difficult to assess in light of the cryptic life history of centipedes of this order. Available data on the centipede fauna of the Indo-Malayan Region are almost exclusively drawn from hand collecting of specimens (Haase 1887, Pocock 1894, Silvestri 1895, Attems 1914, Attems 1930, Verhoeff 1937, Lewis 1991).

The re-discovery of *M.javanica* at a geographically distant new locality and within a new habitat highlights the broad applicability of conclusions drawn from comparison of the efficacy of different centipede sampling techniques in temperate forests. Identifying a hitherto overlooked ecological category at the study site showcases the utility of soil core extraction for collecting rare, small-bodied endogeic centipedes and highlights the importance of a diversified sampling strategy (Baba et al. 2023) for adequately characterising the centipede community of tropical rainforests. The present case reinforces previous conclusions drawn for employing mutliple sampling methods to effectively record euedaphic centipede species (Oliveira et al. 2019, Tulande-M et al. 2020), regardless of taxonomic affinity.

## Supplementary Material

XML Treatment for Mesoschendyla javanica

## Figures and Tables

**Table 1.** Geophilomorph centipedes described from the Sunda Islands and the Malay Peninsula known only from type material and with a presently unrevised taxonomic status.

Family	Species	Locality	Taxon reference
Geophilidae	*Javaeniabataviana* Chamberlin, 1944	Batavia (Jakarta), Java, Indonesia	Chamberlin (1944)
Schendylidae	*Ballophilusconservatus* Chamberlin, 1944	Goenoeng Malabar (Gunung Malabar), Java, Indonesia	Chamberlin (1944)
	*Ballophilussabesinus* Chamberlin, 1944	Sebesi Island, Sunda Strait	Chamberlin (1944)
Mecistocephalidae	*Mecistocephalusmonticolens* Chamberlin, 1920	Gede (Gunung Gede), Java, Indonesia	Chamberlin (1920)
	*Mecistocephaluscelebensis* Chamberlin, 1920	Bua-Kraeng (Gunung Bawakaraeng), Sulawesi, Indonesia	Chamberlin (1920)
	*Mecistocephalusenigmus* Chamberlin, 1944	Poentjak (Puncak), Java, Indonesia	Chamberlin (1944)
	*Mecistocephalusstenoceps* Chamberlin, 1944	Purmerend, Batavia (Jakarta), Java, Indonesia	Chamberlin (1944)
	*Mecistocephalusmalayensis* Chamberlin, 1953	Cameron Highlands, Pahang, Peninsular Malaysia	Chamberlin (1953)
	*Mecistocephalustridens* Chamberlin, 1922	Buitenzorg, Java, Indonesia	Chamberlin (1922)
	*Tygarrupgriseoviridis* Verhoeff, 1937	Brinchang, Pahang, Peninsular Malaysia	Verhoeff (1937)
	*Tygarrupmalabarus* Chamberlin, 1944	Goenoeng Malabar (Gunung Malabar), Java, Indonesia	Chamberlin (1944)
